# Vulnerable Older Adults’ Identification, Geographic Distribution, and Policy Implications in China

**DOI:** 10.3390/ijerph182010642

**Published:** 2021-10-11

**Authors:** Xiaoyi Jin, Yanjun Liu, Zhaoyuan Hu, Wei Du

**Affiliations:** School of Public Policy and Administration, Xi’an Jiaotong University, No. 28 Xianning West Road, Xi’an 710049, China; xiaoyijin@mail.xjtu.edu.cn (X.J.); kuiyj09@163.com (Y.L.); duwei@mail.xjtu.edu.cn (W.D.)

**Keywords:** vulnerable older adults, latent class analysis, geographic distribution, policy implications

## Abstract

With the population aging and urbanization in China, vulnerable older adults tend to show more complex characteristics, bringing great challenges to public health policies. Using China Longitudinal Aging Social Survey data 2014, this paper builds a comprehensive index system for the identification of vulnerable older adults from three dimensions, including health, economy, and social support, then divides older adults into four support levels and six small classes by using the typological method. The results show that older adults in urgent need of assistance or priority are those poor in health and economic conditions, 1.46% of them are highly vulnerable because of the lack of social support; 12.76% of them obtain a certain social support are moderately vulnerable; and 34.72% of them are slightly vulnerable with disadvantage in only one dimension. The geographic distribution of different types of vulnerable older adults varies significantly. The paper provides evidence to design more feasible and specific policies with comprehensive considerations for different types of vulnerable older adults residing in different regions.

## 1. Background

The vulnerable groups are special groups with poverty, low quality of life, and vulnerability of endurance, and are as such defined in terms of poor social status and social circumstances. A direct cause of vulnerability is the lack of personal ability (natural or acquired incapacity), and the deep reason is the social structure flaw [1], namely the unfair social system arrangement. China has the world’s largest elderly population, and faces a rapidly aging process. The proportion of population over 60 years old in China has increased from 10% in 2000 to 18.7% in 2020, and those 65 years old and above in 2020 had reached 190 million [2]. With aging, the proportion of vulnerable older adults, such as solitary individuals [3] and oldest old [4] will continue to rise [5]. According to preliminary analysis of “The Fourth Sample Survey on the Living Conditions of China’s Urban and Rural Older Persons” by China National Commission on Aging, 59.4% of older adults had economic difficulties, 47.0% had health problems in varying degrees, and more than 40 million disabled older adults need priority assistance. The distribution of vulnerable older adults was unbalanced between urban and rural areas and varies among regions, with the proportion of vulnerable older adults significantly higher in rural areas than in urban areas, and higher in the central and western regions than in the eastern regions [6]. China’s antipoverty program has eradicated extreme poverty in 2020 [7] and significantly improved economic conditions of older adults, but they are still at risk of becoming poor due to illness and old age. Therefore, the improvement of public health policies mainly focusing on the medical care system, and the improvement of social support policies related to mental health and active aging, are core issues in the next stage [8]. Social changes such as population aging and population mobility [9] have made older adults the high-risk social group. To effectively improve relevant policies, characteristics of older adults and differences between supply and demand of current public policies in different regions should be fully considered. However, the quantitative analysis on particularity, internal differences, and distribution characteristics of vulnerable older adults are rare, which is detrimental to consolidate the achievements of anti-poverty governance and will restrict the foreseeable overall design of public health policies.

Compared with young adults, older adults have a great decline in physiological functions, are less functional in the family, and are more likely to rely on others or society for self-care and livelihood. Vulnerable older adults usually have no economic income, low political influence, high psychological pressure, and a strong sense of social alienation [10]. There are different types of vulnerability for elderly adults. Previous studies mostly identified vulnerable older adults based on part of their vulnerable dimensions, such as individual/family characteristics, health status, economic level or others, and focused on special groups such as women, the aged, those living alone/empty nest, the disabled, the poor, and older adults who lack community services. Some studies also focused on the care needs and social assistance for older adults with “double difficulties” (difficulties in economy and self-care) from the perspective of economic and health [11]. With aging and urbanization, the traditional vulnerable older adults, such as women, the aged, living alone/empty nest, the disabled, and the poor older adults, not only continue to expand, but also show new characteristics and superimposing vulnerability, making it more difficult to design a diversified accurate old-age assistance system. Accordingly, it is necessary to identify vulnerable older adults more precisely, which helps to understand their demand from a policy perspective.

Research on social vulnerability have found that social transition in China has created new vulnerable groups [12]. Among them, older adults, such as left behind women [13] and childless individuals [14], have to suffer higher vulnerability. Most of the research focus on conceptual definition and situation analysis of some special older groups in a certain area, such as left behind women in rural China [13] or urban older adults in poverty [15], trying to put forward reasonable proposals through sufficient qualitative description. The urban and rural older adults are distinguished by hukou, namely the household registration type, which induces the differences in social security, welfare system, and medical resources between urban and rural residents. Accordingly, it is necessary to conduct a comparative analysis of the older adults with different hukou types. While research on overall elder adults with rural or urban hukou by using quantitative methods to measure social vulnerability are seldom seen, this paper aims to construct a comprehensive identification system for the vulnerable older adults, which could accurately reflect basic characteristics of older vulnerable groups in urban and rural China. It is helpful to reduce the negative impact of health and support deficit on vulnerable older adults, which is the key issue after the eradication of extreme poverty [7]. It also provides practical evidence and policy recommendations for health and happiness improvement of older adults in the process of population aging.

## 2. Literature Review

### 2.1. Vulnerability of Older Adults

Vulnerability as an analytical concept first emerged in the environmental sciences, specifically for the study of human impacts of natural disasters [16]. Vulnerability in disaster studies was initially defined as the “potential for disruption or harm” [17]. In old-age studies, the term “vulnerable” was often employed as an ill-defined descriptor of people or groups who are in some way disadvantaged, or as a euphemism for “poor”, “dependent”, “frail”, or “isolated” [18,19,20]. Vulnerability in older adults has mainly been approached by identifying high risk groups with disadvantages, such as the poor, childless, frail, isolated [16], the very old, and those with limited opportunities or capacities to exercise autonomy [21]. While it is certainly possible to study all the disadvantage factors, this “one thing at once” approach is limited, especially for older adults in whom complex sets of social circumstances may exist and interact in different (possibly unpredictable) ways to contribute to vulnerability in an aggregate sense [22]. Vulnerability is the outcome of complex interactions of risks, and recent work on the quantification of frailty may provide a guide to quantify social vulnerability. Social vulnerability is the result of complex interaction of multiple risks. Recent quantitative studies on frailty provide some reference and guidance for the analysis of social vulnerability [23,24]. When people get older, they are often confronted with health deficits, become frailer, and have increasing risk of poor health outcomes and mortality [25,26].

Frailty is a concept that describes vulnerability to adverse health outcomes as a result of decreased reserve capacity and resistance to stressors [27]. The concept of frailty has developed from a perspective that emphasizes physical aspects of frailty [28] to a more integral perspective that comprises the multidimensional aspects of frailty [29,30,31]. It may be regarded as a dynamic state that affects an individual experiencing loss in one or more areas of human functioning [32], and it increases with the accumulation of physical, functional, cognitive and psychological problems with memory and attention, reduced vision or hearing, and social deficits, i.e., “deficit accumulation” [33]. Frailty has been widely used as an index to measure the comprehensive health status of older adults [34].

Although individuals of all ages are potentially vulnerable to social disadvantages, social disadvantages are particularly important for older adults [35]. In the context of aging, as many older adults cannot cope with the impact of social and economic changes on themselves, it has resulted in social vulnerability on themselves [36]. On the one hand, many studies have confirmed that social environment can affect the health of old adults; on the other hand, the death of relatives and friends leads to the shrinkage of the social network of older adults with aging, and the decline of health also brings difficulties to the participation of older adults in social activities [37]. The increasing prevalence of social vulnerability in aging societies is incontrovertible [36]. If the cumulative method of deficit in the frailty study is applied to the analysis of social vulnerability, it can provide a new way to understand the complex health needs and social nursing needs of older adults [22]. Therefore, Andrew proposed that a social vulnerability index, similar to the Frailty Index, can comprehensively measure the overall social status of older adults [22,36].

### 2.2. The Indicators Identifying Vulnerable Older Adults

Given the complexity of social environment, many social factors, including socioeconomic status (SES), deprivation, social support, social isolation or exclusion, social networks, social engagement, mastery and sense of control over life circumstances, social capital, and social cohesion are likely to affect health of older adults [35]. One method of operationalizing social factors influencing health is to combine them into the concept of “social vulnerability” [22], which can be broadly understood as the degree that a person’s overall social situation leaves he/she susceptible to health problems. Social vulnerability can be considered as influences from individual to family, wider networks, and societal context at various levels [35]. The prior studies have illustrated the cumulative effects of the lack of social engagement, social companionship, social support and poor socioeconomic status [36]. The concept of social vulnerability provides a holistic quantification of social vulnerability among older adults and appears to be a valid measure [38,39].

As social vulnerability is a multifactorial and multilevel quantification of a person’s social environment [22,40], the reliance on a single factor is likely to lead to misclassification; therefore, holistic, integrative, and comprehensive approaches to measure social circumstances are desirable [39,40]. Andrew believed that the human ecology perspective, originally proposed by Bronfenbrenner (1979) [41], provided the initial conceptual framework, including physiology, behavior, material, and psychology aspects, for study of social vulnerability among older adults [35]. Up to now, there are only a few empirical studies on social vulnerability, which mainly focus on the relationship between social vulnerability and mortality. Due to the differences in socioeconomic development and political system in various countries and different data used by various research, dimensions measuring social vulnerability vary significantly (see Table 1).

Table 1 shows that: Firstly, Andrew accumulated achievements in measuring the social vulnerability of older adults and tried to build a comprehensive social vulnerability index at the beginning of his study based on previous relevant research and data accessibility [22,38]. In the follow-up study, he adjusted the index in consideration of social relations and scientifically used factor analysis to identify dimensions of social vulnerability [35].

Secondly, although social vulnerability had been constructed differently in the previous research, several estimates were closely replicable. This suggests that the social vulnerability index has potentially wide applicability: The constituent variables vary with settings as long as the basic tenet including multiple social factors relating to important broad domains is met [22]. The measurement of social vulnerability in existing related research basically includes the dimensions of economic level, social support, and social participation.

Thirdly, most of previous studies focused on the relationship between social vulnerability and health status of older adults. To measure social vulnerability, overall social status was more concerned instead of health indicators. Only a few studies added mental health factors in constructing indicators, for example, Ryff scales, feeling about life [22,27,38], loneliness [39], and mental situation [34]. Health factors, as one of important human capital of older adults, should be used to construct a more comprehensive measurement of vulnerability.

Up to now, social vulnerability has only been used in a few empirical studies since the variables constructing social vulnerability probably change significantly among countries or the connotation of the same variable may be different [34]. Older adults in China are quite distinctive for its numerous population size, rapid growth rate, and obvious regional and rural–urban differences [43]. Their economic needs have been basically concerned by social security system, but their vulnerability related to health and social participation has not been carefully considered. Therefore, a comprehensive measurement of the vulnerable older adults in China needs to be constructed, which could be helpful to analyze how to achieve stable health improvement of older adults under rapid aging, as well as explore the improvement of happiness of vulnerable older adults after the elimination of extreme poverty [7].

## 3. Data and Methods

### 3.1. Data

The data come from China Longitudinal Aging Social Survey (CLASS) undertaken by Renmin University of China in 2014, which is one of the most representative national data of older adults in China (http://class.ruc.edu.cn for more information about the CLASS 2014. accessed on 2 October 2021). The survey was conducted using a stratified multi-stage probabilistic sampling method. The county/district was the primary sampling unit (PSU), the village/neighborhood committee was the secondary sampling unit (SSU), and population aged 60 and above were chosen as respondents in the use of drawing sampling method in each village/neighborhood committee. The weighting was designed by population and households in each unit and adjusted according to actual implementation and number of older adults in each family. The survey included older adults’ personal characteristics, health and related services, socioeconomic statue, pension planning and social support, cognitive and aging attitude, and their relations with children, etc. Thus, the data provide sufficient information of vulnerability indicators, individual and social characteristics of older adults in mainland China. The samples include 11,511 individuals aged 60 and above, which were collected in 28 provinces or regions. Provinces or regions not sampled are Hong Kong, Taiwan, Macao, Hainan, Xinjiang and Tibet, which are either Special Administrative Regions or remote autonomous regions for ethnic minorities or islands. Considered the feasibility of the survey implementation, the CLASS survey is a representative national survey of older adults in mainland China. The overall age distribution was similar with that of the 6th China population census in 2010 [44].

### 3.2. Method of Analysis

First, according to the previous research (see Table 1) and data accessibility of CLASS 2014, a preliminary selection of the identification indicators of vulnerable older adults was made. Exploratory factor analysis was performed on those operable variables. Factor analysis can represent potential variables by using the linear function relations with less common factors and the sum of some specific factors. Its core idea was to reduce dimensions, namely some complex and correlated variables could be combined into one or a few irrelevant “factors” which reflect certain characteristics [45]. By using factor analysis method, this paper constructed a set of identification index system of vulnerable older adults.

Previous studies mostly measured the comprehensive social situation of older adults by calculating social vulnerability index without identification and classification of the vulnerable types, which makes it difficult to reveal characteristics and vulnerable status of older adults. Therefore, in the second step, Mplus software (Muthén & Muthén: Los Angeles, CA, USA) and latent class analysis (LCA) were used to identify the vulnerable older adults in China. LCA is a statistical method that analyzes categorical latent variables that are beyond the categorical variables, and it can effectively use different index of vulnerability to generate various types of vulnerable older adults.

Third, through the cross-table analysis, this paper revealed group characteristics and distribution of different types of vulnerable older adults in order to provide evidence for solutions of precise assistance for vulnerable older adults.

## 4. Identification of Vulnerable Older Adults

### 4.1. Identifying Variables

The measurement of social vulnerability is multi-dimensional and multi-index. Existing research generally incorporate economic status, social support, and social participation. Because of the limitation of specific research purpose, most of the studies only included mental health indicators without considering the factors of physical health. However, as one of important human capital of elder adults, physical health is particularly important in measurement of vulnerability. Therefore, this paper regarded health factors and social factors as two important dimensions to identify vulnerable older adults, and attempted to construct a comprehensive index system for the identification of vulnerable older adults. The index system covers four aspects: health status, socioeconomic status, social support, and social engagement. Considering data accessibility of CLASS 2014, the following 12 variables are selected to classify and identify the vulnerable older adults (Table 2).

Health status. Health status includes health self-assessment, Barthel index, and chronic and mental health. The health self-assessment was coded from 5 to 1 according to the level of health from high to low. Barthel index was measured by the activities of daily life (15 = no difficulty, 10 = few difficulty, 5 = some difficulty, 0 = cannot do) in 10 performing tasks. The Barthel index ranged from 0 to 100, and the lower the score, the more serious the health problem. Chronic is measured through “What chronic diseases do you have?” as a continuous variable. On the “Did you feel sad in the past week?” deficit, which includes three response categories, scores are 1 if the answer is “No”, 2 if the answer is “Sometimes”, and 3 for “often”.

Socioeconomic status. Socioeconomic status contains income, economic independence, and housing property. Income is measured through “in the past 12 months, what is your personal total income?” as a continuous variable. Economic independence is measured through “What is your main source of income?”. If the main source of older adults’ income is their or their spouse’s pension or labor payment, this variable is coded as “1”; Otherwise coded as “0”. As housing property today represents great wealth of older adults, it can reflect the socioeconomic situation of older adults, which is measured through “How many houses do you (and your spouse) have?” as a continuous variable.

Social support. Social support includes social network support and children’s economic support. Social network support includes three variables: someone that older adults meeting/contacting, discuss personal affairs, or look for help. Children’s economic support is assessed by monetary or material assistance within the past 12 months.

Social engagement. Social engagement contains community security patrols, caring for other older adults, caring for children, environmental protection, dispute mediation, chatting, volunteer service, and others. This paper measures the number of activities older adults had participated in the past 12 months as a continuous variable.

### 4.2. Identifying Indicators of Vulnerable Older Adults: Factor Analysis

Identifying indicators of the vulnerable older adults is studied by factor analysis method. Some multi-category variables are re-coded quantitatively and standardized, by assigning values of each option according to the degree and the direction of “social deficits”. For example, the answers of “How do you feel about your current health status? (1) Very healthy, (2) Relatively healthy, (3) General, (4) Relatively unhealthy, and (5) Very unhealthy” are assigned 1–5 respectively. In the process of standardization, the MIN-MAX range standardization method is used to unify the variable range between [0,10] intervals, eliminating the dimensional influence of the original variables, and the influence of the variation size and the numerical size.

To test data, the rationality and sampling adequacy of factor analysis, the Kaiser–Meyer–Olkin test and the Bartlett test were used. A KMO value of 0.713 (>0.5) revealed the sufficient sampling, and a significance level from the Bartlett test <0.01 indicated that the data are appropriate and useful to substantially reduce data dimension. Then, three common factors with eigenvalues >1, as the eigenvalue of those three is 2.34, 1.71, and 1.43 respectively, are extracted for the varimax-rotated analysis. The three common factors explained a tremendous 45.74% of the total variance, and it is reasonable to select them as the main factors (VF1, VF2, and VF3).

Table 3 shows that the variables with factor loading up to 0.5 is very suitable and is selected into the Factors. Specifically, VF1 is largely composed of “someone to meeting/contacting”, “someone to discuss personal affairs”, and “someone for help” which represent social support. VF2 has strong positive loading on “health self-assessment”, “Barthel index”, and “chronic”, suggesting the health status. VF3, implying the socioeconomic status, consists of the “income”, “economic independence”, and “housing property”. The social engagement weighted on leisure activities failed to form one VF.

### 4.3. Typologies of Vulnerable Older Adults: Latent Class Analysis

All the identification indicators of the vulnerable older adults were treated as binary variables, and the responses to each item were assigned a value of “1” if representing a deficit and “0” otherwise. Table 4 reports distribution of items in different dimensions. Firstly, social support included three variables, persons whom older adults meet/contact, discuss personal affairs, or look for help. It was scored 0 if he/she had potential support from family members, relatives, or friends on the above aspects, and 1 if he/she did not have. Secondly, health status included health self-assessment, Barthel index, and chronic. Health self-assessment was coded as 0 = healthy, 1 = unhealthy. The Barthel index was used to determine whether older adults rely on others for life-care (0 = no, 1 = yes). Chronic was coded as 0 = no chronic disease, 1 = have chronic disease. Thirdly, economic status included income, economic independence, and housing property. Income was coded as 0 = higher than older adults’ average income, 1 = lower than older adults’ average income. Economic independence was coded as 0 = the main income resource is older adults themselves, 1 = the main income resource is not older adults themselves. Housing property was coded as 0 = owning property, 1 = no property.

We employed Mplus to analyze the response patterns generated from the cross-classification of dichotomous indicators of vulnerable older adults. The latent class model with only a single class was used as the baseline model, and then successively added the number of classes in order to determine the optimal model by checking for model fit [46]. The goodness-of-fit measures used to select the optimal models were the Akaike information criterion (AIC), Bayesian information criterion (BIC), and sample size-adjusted BIC (aBIC). The above fitness was useful for selecting the best-fitting model among reasonable but competing models, with a smaller value of that fitness providing the better model fit [47]. The entropy index, which ranged from 0 to 1, is often used to evaluate the accuracy of classification in LCA. When entropy was 0.6, it showed that about 20% of individuals had classification errors, while when entropy equaled 0.8, it showed that the accuracy of classification exceeded 90% [48]. Mplus also provided the Lo–Mendell–Rubin likelihood ratio test (LMR), and the significant *p*-value of the LMR indicated that the model with more classes fit significantly better [47].

Table 5 reports the goodness-of-fit statistics for seven models of vulnerable older adults. For all seven models, the LMR were significant. But in the first six models, the BIC decreased successively with each additional class added, indicating relative improvements in model fit; while when the classification reached seven, the value of BIC increased, indicating that the six-class model was the fittest.

Table 6 shows the conditional probabilities and latent class probabilities of the six-class model of vulnerable older adults, which helps to facilitate the observation and comparison, and find out more intuitively the prominent vulnerable characteristics of different types of vulnerable older adults.

Table 7 shows the characteristics and proportion of different types of vulnerable older adults. Based on these patterns, elderly adults were divided into four levels: high vulnerability (need assist urgently), moderate vulnerability (focus group), slight vulnerability (care group), and potential vulnerability (vulnerability prevention). According to the characteristics, elderly adults were further divided into six subtypes: (1) multi-vulnerable (1.46%), (2) dual-vulnerable (12.76%), (3) support-vulnerable (1.34%), (4) economy-vulnerable (14.21%), (5) health-vulnerable (19.17%), and (6) potential-vulnerable (51.06%). Each subtype shows a specific demand for social assist, which reveals a more explicit policy implication. First, highly vulnerable older adults are poor in both health and socioeconomic status without close relatives or friends; thus, they could neither take good care of themselves nor ask someone for help. As a result, they are in urgent need of policy assistance. Second, compared with highly vulnerable older adults, moderate vulnerable ones could get some help from their family or friends, so their life is tough but still relatively sustainable. Their plight reflects shortcomings of current public policies, so they are the key objects of policy design and improvement. Third, there are three kinds of slightly vulnerable older adults facing only one kind of vulnerability, such as poor, illness, or loneliness. These older adults are not so frail currently, but may become vulnerable in the near future when they get older and if the public service resources are still limited or the social support pressure increases. Finally, potentially vulnerable older adults are relatively good in all aspects up to now, but it does not mean that they are inessential for public policy improvement. Older adults are inherently vulnerable groups compared with the young, and their vulnerability deepens as they grow older. Therefore, to design forward-thinking public service policies, their demands would be the main basis.

### 4.4. Characteristics of Different Types of Vulnerable Older Adults

Gender differences. Compared with males, vulnerability of female older adults is relatively high. Males, in particular, are more likely to suffer shortage of social support, and females are more likely to suffer shortage of economic support and deteriorating health in older age.

Age distribution. With the aggravation of vulnerability, the proportion of the oldest old is increasing. Vulnerability is basically caused by the lack of social support for the younger older adults.

Marital status. Older adults with no spouse are more likely to face the dual vulnerability in terms of economy and health. While among the support vulnerable older adults, the proportion of older adults with no spouse is much lower. It comes probably because older adults with no spouse are more likely to get social support from others.

Living arrangement. Compared with other types of vulnerable older adults, the proportion of support vulnerable older adults living alone is relatively high, followed by the multi-/dual-disadvantaged older adults.

Social insurance. Participation in social security plays the role of economic support to a certain extent, and helps to prevent older adults from becoming increasingly vulnerable.

Table 8 shows the characteristics difference of household registration among vulnerable older adults. Among the multi/dual vulnerable older adults, compared with NA-older adults, the proportion of the A-older females was lower, and that of older adults without social insurance was higher. However, among the support vulnerable older adults, compared with NA-older adults, the proportion was higher for A-older females and lower for those without social insurance. Among the economic vulnerable older adults, compared with the A-older adults, the proportions of females and non-spouse ones were relatively high in NA-older adults. For the health vulnerable older adults, compared with the A-older adults, the proportions of older adults with older age, no spouse, and no insurance were relatively high.

## 5. Geographic Distribution of Vulnerable Older Adults

### 5.1. Classification of Provincial Economic Development and Population Aging

Besides demography, economics is another important subject to explain the causes of population aging and regional differences. A causal relationship between economy and population has been widely accepted in the economic academia [49]. At present, many Chinese scholars have analyzed the relationship between population aging and economic development. Most of the studies proposed that there is a significant correlation between population aging and economic level [50,51]. For example, some scholars use provincial data to confirm the correlation between GDP per capita and aging coefficient [52,53,54]; and some scholars believe that there is a long-term stable relationship between per capita consumption, GDP per capita, and the ratio of population aging [55], etc.

Population aging is affected by many factors, including population development, economic factors, social factors, etc. With the development of social economy, natural environment has little and fixed influence on population aging, while the economic, as the main structural factor, has a significant impact on the evolution of the aging pattern [56]. GDP per capita represents the level of social and economic development of the region. The improvement of this index is conducive to the improvement of people’s living quality and welfare, and the acceleration of aging [57]. Therefore, GDP per capita and regional aging rate were used to analyze the distribution law between macro-economic indicators and vulnerable older adults.

**Figure 1:**
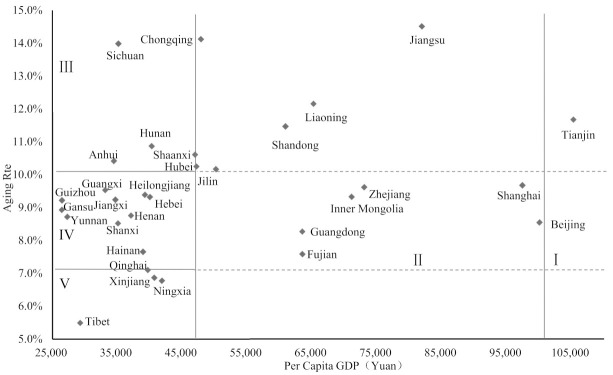
Classification of provincial economic development and population aging.

The provincial GDP per capita (Data source: https://data.stats.gov.cn/easyquery.htm?cn=E0103, accessed on 2 October 2021) and aging rate (Data source: https://data.stats.gov.cn/easyquery.htm?cn=E0103, accessed on 2 October 2021) are set as the horizontal and vertical coordinates respectively in Figure 1. When making the quadrant, this paper took into account the GDP per capita and the aging rate (10.06%) in 2014. The quadrant was divided by the national GDP per capita in 2014 (47,005 yuan (Data source: http://data.stats.gov.cn/easyquery.htm?cn=C01&zb=A0201&sj=2015, accessed on 2 October 2021) and 100,000 yuan GDP per capita (solid lines on the left and right sides of the horizontal axis), the aging rate of 10% and 7% (solid lines on the top and bottom of the vertical axis). China’s provinces and regions were divided into five categories (Figure 1):

(1) Super high–high area. Beijing, Tianjin, and Shanghai are the typical areas. The main characteristics are “super high GDP per capita, high aging rate”. Because economic development has enriched people’s food structure, improved living condition and promoted development of health service, economic growth prolonged local life expectancy and increased the proportion of elderly adults [53]. The super high level of regional economic has indeed greatly promoted the process of aging.

(2) High-multistage area. The representative provinces are Jiangsu, Liaoning, Shandong, Zhejiang, Inner Mongolia, Fujian, and Guangdong. The GDP per capita of these regions is higher than the national average. However, these regions are at different aging stage, and the level of aging is quite different. Population mobility also contributes to the formation of regional differences in aging in China. Because of expanding scale of rural-urban migration and continuing low birth rate, Liaoning suffers more serious aging than other provinces [58]; while in Guangdong, the large immigration population greatly weakens local aging degree [59], showing a significant “dilution effect” [60].

(3) Medium–high area. The main characteristics are “medium GDP per capita, high aging rate” for Chongqing, Sichuan, Hubei, Hunan, Anhui, Jilin, and Shaanxi. In these provinces, the GDP per capita is slightly lower but the aging rate is higher than the national average level. The reason of aging in Sichuan and Chongqing is different from Liaoning as elsewhere in Northeast China: The phenomenon of outmigration in Northeast China can be found at all ages, while in Sichuan and Chongqing it only exists at a working age [61,62].

(4) Low–medium area. The main characteristics are “low GDP per capita, medium aging degree” for Henan, Hebei, Heilongjiang, Guangxi, Jiangxi, Guizhou, Gansu, Shanxi, Yunnan, and Hainan Provinces. They are transiting from aging stage I (7% < PA < 10%) to stage II (10% < PA < 14%) [60,63], and the GDP per capita and aging degree are slightly lower than national average. These provinces do not have advantaged economic projects and there is a strong correlation between economic development and population aging.

(5) Low–low area. Tibet, Xinjiang, Qinghai, and Ningxia are the representative provinces with characteristics of “low GDP per capita, low aging rate”. These areas are still at adult type stage II (5.5% < PA < 7%) or just entering aging stage I (7% < PA < 10%) [60,63]. The economic development of these areas is backward, and the degree of aging is far below the national average. There is a low correlation between economic and aging, and the main reasons include: These provinces contain a large number of ethnic minorities. Local attitude towards reproduction and loosen fertility policies for minorities lead to the high birth rate. Besides, as the underdeveloped health service and harsh natural environment does harm to health, the life expectancy of older adults is low [64,65].

### 5.2. Distribution of Vulnerable Older Adults in Different Areas

**Figure 2:**
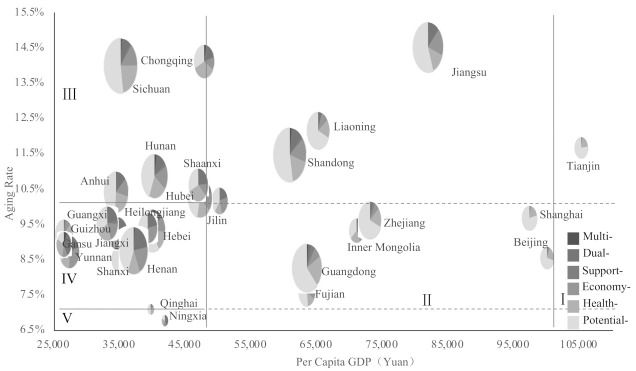
Distribution of vulnerable older adults in different areas. Note: To avoid overlap, the bubbles are compressed to ensure that more areas can be exposed. The multi-vulnerable group is actually bigger than the slice looks.

Figure 2 shows the relationship between provincial distribution of vulnerable older adults and core macro-indicators, and the bubble size represents the scale of aging population in each area. Overall, with GDP per capita increasing, the proportion of potential vulnerable older adults gradually increases. (1) In super high–high areas, the proportion of potentially vulnerable older adults was as high as 70%, while the proportion of multi/dual ones was only about 3%. (2) In high–multistage areas, the proportion of potentially vulnerable ones was basically over 50%, and the proportion of multi/dual older adults was about 5–15%. However, in Inner Mongolia, the proportion of potentially and multi-/dual-vulnerable older adults were both about 30%. (3) In medium–high areas, the potentially vulnerable older adults accounted for about 30–50% in each province, and the multi-/dual-vulnerable older adults accounted for about 10–20%. (4) In low–medium areas, such as Guizhou, the potentially vulnerable older adults exceeded 68%, and the multi-/dual-vulnerable older adults were only about 3.3%. In Shanxi and Hebei, the potentially vulnerable older adults exceeded 55%, and the multi-/dual-vulnerable older adults accounted for 11%. The potentially vulnerable older adults in Jiangxi, Henan, Guangxi, and Yunnan accounted for about 30–40%, and the multi-/dual-vulnerable older adults accounts for about 20%. In Gansu, the proportion of potentially vulnerable older adults was less than 18%, and that of multi-/dual-vulnerable older adults was more than 41%. (5) In low–low areas, the proportion of potentially vulnerable older adults in Qinghai was more than 50%, and that of multi-/dual-vulnerable older adults was about 10%, while the proportion of potentially vulnerable older adults in Ningxia was less than 20%, and that of the multi-/dual-vulnerable older adults was more than 42%.

**Figure 3:**
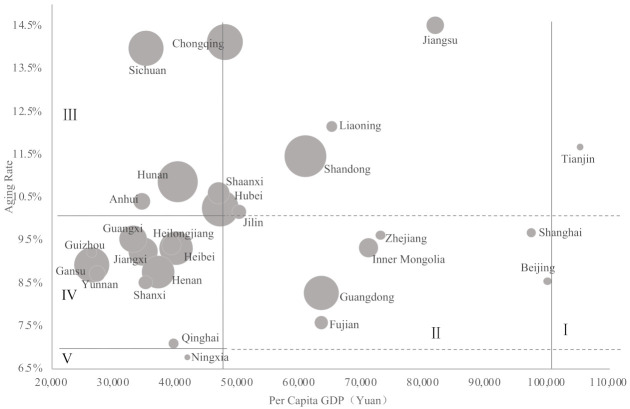
The scale of multiple vulnerable older adults in different areas.

In Figure 3, the bubble size represents the size of the multiple vulnerable older adults in each province. There were significant regional differences in scales of the highly vulnerable older adults. (1) In super high–high areas, there were fewer than 10 thousand multi-vulnerable older adults in each province. (2) In high–multistage areas, the number of multi-vulnerable older adults in each province was about 15–40 thousand. However, in economically developed regions, such Guangdong and Shandong, because of the larger size and high proportion of multi-vulnerable older adults, the scale of multi-vulnerable older adults was relatively large (155 thousand, 227 thousand, respectively). (3) In medium–high areas, there were about 30–60 thousand multi-vulnerable older adults in Jilin, Anhui, and Shaanxi, and about 150–200 thousand in Sichuan, Chongqing, Hubei, and Hunan. The differences in scales of the multi-vulnerable older adults were mainly caused by the size of older adult population and proportion of multi-vulnerable older adults. (4) In low–medium areas, there was only about 10–30 thousand multi-vulnerable older adults in the economically backward provinces, such as Guizhou, Shanxi, Yunnan, while the economy of Gansu was poor, and the scale of multi-vulnerable older adults was as high as 150 thousand or so. There were about 100–150 thousand multi-vulnerable older adults in the developed provinces. Although Heilongjiang had a relatively high level of economy, the scale of multi-vulnerable older adults was less than 50 thousand. (5) From the perspective of low–low aging areas, the scale of multi-vulnerable older adults in Ningxia and Qinghai was only about 10 thousand, which may have been caused by the poor natural environment, low medical level, and relatively high mortality rate of older adults.

## 6. Conclusions and Policy Implication

By using the latent class analysis method, this paper identifies the vulnerable older adults in urban and rural areas of China in terms of health status, socioeconomic status, and social support, and classifies older adults into six vulnerable types, corresponding to four levels of assistance urgency. The group characteristics and geographic distribution of different vulnerable groups were also analyzed, which provides evidence for policy makers to reduce vulnerability of older adults integrally.

First, in the context of aging and urbanization in China, the characteristics of disadvantaged older adults became more complex and diverse. This paper revealed six types of vulnerable older adults, i.e., the most vulnerable older adults facing high deficit accumulation (multi-vulnerable), the most common vulnerable older adults with health and economic disadvantages (dual-vulnerable), and older adults with a certain advantage (potentially vulnerable), as well as the support, economy, or health vulnerable older adults based on one disadvantage. The results showed that 1.46% of older adults had high vulnerability, 12.76% had moderate vulnerability, 34.72% had slight vulnerability, and about one half of older adults (51.06%) were in the potentially vulnerable group, which is highly consistent with the results of data from “The Fourth Sample Survey on The Living Conditions of China’s Urban and Rural Older Persons” [6], which confirms the reliability of the typology analysis method and the type identification of the vulnerable older adults in China is robust. From the prospective of public policy, the identification of vulnerability based on single dimension was not convincing since older adults who are at higher risk of vulnerability usually suffer from multi-dimensional vulnerability, interacting with health, socioeconomic, or social support factors. Therefore, in the context of limited medical resources, unbalanced regional development and incomplete health policies, a multi-dimensional and multi-standard identification index system for the vulnerable older adults is necessary for policy makers, which helps to more accurately evaluate vulnerability and design more specific policies to improve welfare of older adults with high efficiency and low cost.

Second, identifying characteristics of different types of vulnerable older adults helps to improve effectiveness of policies. Among females, aged, widowed and nest older adults, the proportion of multiple vulnerable older adults was higher, as was the degree of vulnerability. Specifically, female and widowed older adults were more vulnerable in terms of health and economy, while male and young older adults were more likely to lack social support. From the prospective of social insurance, the health condition was closely related to socioeconomic status, as social insurance could prevent older adults from economic plight and multi-/dual-vulnerable to a certain degree. Speeding up and perfecting the social insurance system is of great practical significance for improving the living welfare of older adults. To improve further relevant policies, especially public health policies, characteristics of different vulnerable older adults should be fully considered: (1) taking advantages of social insurance security to meet basic demands of older adults with high/moderate vulnerability; (2) formulating precise support policies to promote diversified old-age care model for different types of vulnerable older adults; (3) referring to the framework of Active Aging and Healthy China policy, the development needs of the potentially vulnerable older adults can be met, and the overall improvement of older adults’ welfare can be realized.

Third, due to the population mobility and unbalanced allocation of medical resources, urban and rural differences were significant among vulnerable older adults in China. The distribution of different types of vulnerable older adults varied greatly. Among the support vulnerable older adults, male and agricultural household registration older adults accounted for a higher proportion. This may be because, on the one hand, female older adults are more likely to have social relations (such as brothers and sisters, friends, groups, etc.) [66], while males are more likely to face the lack of social relations. On the other hand, with more and more rural labors migrating to cities for higher-paid job, social support from young adults to rural older adults has been greatly weakened since out-migration of adult children has destroyed traditional extended family in rural China.

In addition, health vulnerable older adults with urban hukou are significantly different from those with rural hukou, which is caused by the imbalance of medical resources allocation between urban and rural areas. The urban older adults have better social security support and more abundant medical resources, which guarantees their health and welfare in their later years. However, the medical conditions in rural areas are relatively backward and the accessibility of medical services is poor, which leads to the rural older adults usually suffer from a higher health dilemma and higher death risk. It is urgent to ensure health status of rural older adults by vigorously developing rural economy, continuously promoting rural revitalization and promoting rural medical care.

Fourth, local policies should be suitable for their own reality in terms of economy, degree of aging and stock of vulnerable older adults. According to the indices of economic development and population aging, China’s provinces can be roughly divided into five types: super high–high area, high–multistage area, medium–high area, low–medium area, and low–low area. The distribution of vulnerable older adults in different areas varies greatly. On the whole, with regional economic level increasing, the proportion of potential vulnerable older adults gradually increased, while the multiple-/dual-vulnerable older adults gradually decreases, and the scale of multiple vulnerable older adults gradually reduces. Government departments should comprehensively assess the scale of vulnerable older adults in various areas, and ensure that they can support and deal with the plight of aging accurately.

Specifically, super high–high areas should prevent the deterioration of the disadvantage for potentially vulnerable older adults, and provide one-to-one assistance to the highly vulnerability older adults. In high–multistage and medium–high areas, it is necessary to formulate targeted support policies for older adults with high vulnerability, strengthen the construction of social insurance system, and provide full play to the most critical role of social security. In low–medium areas, the government needs to account the specific situation of various types of vulnerable older adults, and different assistance strategies should be formulated according to the local conditions. For low–low areas, although the aging degree is still low and the scale of vulnerable older adults is small, the governments should be aware of local degree of aging, and make a sound social support policy in advance.

## 7. Limitations

Considering data accessibility of CLASS 2014 and operability of appropriate method, this paper has some limitations. First, the question “Did you feel sad in the past week” was more suitable to measure emotion rather than mental health. While given that there is no alternative index measuring mental health in the CLASS 2014 survey, it was selected to indirectly measure mental health. Second, by using the latent class analysis, this paper recoded the variables as a lot research did [67,68], which might lead to the loss of part of information.

## Figures and Tables

**Table 1:** Dimensions of vulnerability index measurement.

Authors	Data	Dimensions
Andrew, Mitnitski, and Rockwood 2008 [22]	National Population Health Survey (NPHS)	Socio-economic status, social support, social engagement, communication to engage in wider community, living situation, empowerment and life control, and including 23 indicators.
Andrew, Mitnitski, and Rockwood 2008 [22]; Andrew and Rockwood 2010 [37]; Bunt et al. 2017 [27]	Canadian Study of Health and Aging (CSHA)	Socio-economic status, social support, leisure activities, communication to engage in wider community, living situation, socially oriented activities of daily living, Ryff scales, how do you feel about your life in term of…, and including 40 indicators.
Andrew, Fisk, and Rockwood 2011 [22]	Canadian Study of Health and Aging (CSHA)	Socio-economic status, social support, social engagement, living situation, mastery, and including 39 indicators.
Andrew and Keefe 2014 [35]	National Population Health Survey (NPHS)	Socio-economic status, social support, engagement, relations with others, living situation, sense of control, self-esteem, and including 26 indicators.
Armstrong, Andrew, Mitnitski, Launer, White, and Rockwood 2015 [42]	Honolulu-Asia Aging Study (HAAS)	Social support, social engagement, living situation, marital status, and including 19 indicators.
Wallace et al. 2015 [39]	Survey of Health and Retirement in Europe (SHARE)	Social engagement, interpersonal conflict, level of education, loneliness, and including 32 indicators.
Yang and Gu 2017 [34]	China Longitudinal Aging Social Survey (CLASS)	Socio-economic status, social support, mental situation, housing, health service, and including 11 indicators.

**Table 2:** Characteristics of items for the identification of vulnerable older adults (N = 11,511).

Variable		Range	M (SD)
Health status			
X1	Health self-assessment	1–5	3.21 (1.11)
X2	Barthel index	0–100	96.53 (11.81)
X3	Chronic	0–16	1.72 (1.73)
X4	Mental health	1–3	1.37 (0.60)
Socioeconomic status			
X5	Income	0–96,000	18,057.79 (23,686)
	ln (Income + 1)	0–13.77	8.82 (2.10)
X6	Economic independence	0–1	0.76 (0.43)
X7	Housing property	0–12	0.92 (0.55)
Social support			
X8	Someone to meeting/contacting	0–18	7.68 (4.54)
X9	Someone to discuss personal affairs	0–18	4.61 (3.94)
X10	Someone for help	0–18	6.06(4.28)
X11	Financial support from children	0–60,000	3760.92 (5102.57)
	ln (Financial support from children + 1)	0–11.00	6.37 (3.27)
Social engagement			
X12	Participate in leisure activities	0–6	0.33 (0.64)

**Table 3:** Factor load matrix.

	Dimension and Variable	F1 Social Support	F2 Health Status	F3 SE Status
Factors				
Health status				
X1	Health self-assessment	0.103	0.765	0.099
X2	Barthel index	0.044	0.550	0.054
X3	Chronic	0.010	0.752	0.037
X4	Mental health	0.101	0.485	0.201
Socioeconomic status				
X5	Income	−0.001	0.083	0.688
X6	Economic independence	0.064	0.036	0.743
X7	Housing property	0.014	0.065	0.524
Social support				
X8	Someone to meeting/contacting	0.855	0.045	0.021
X9	Someone to discuss personal affairs	0.843	0.042	0.033
X10	Someone for help	0.886	0.042	0.010
X11	Financial support from children	0.189	0.078	0.247
Social engagement				
X12	Participate in leisure activities	0.227	0.046	0.137
Eigenvalue		2.341	1.713	1.436
Variance		0.195	0.143	0.120
Total variance		0.458		

**Table 4:** Distribution of items measuring multiple dimensions of vulnerable older adults (N = 11,511).

Indicator of Vulnerable Older Adults		%
Social support		
X8	Someone to meet/contact	1.50
X9	Someone to discuss personal affairs	12.00
X10	Someone for help	4.52
Health status		
X1	Health self-assessment	28.18
X2	Barthel index	19.00
X3	Chronic	74.89
Socioeconomic status		
X5	Income	58.25
X6	Economic independence	24.47
X7	Housing property	16.42

**Table 5:** Model fitting of vulnerable older adults.

No. of Classes	AIC	BIC	aBIC	Entropy	LMR
1	75,459.050	75,523.698	75,495.097	-	-
2	71,665.003	71,801.482	71,741.103	0.612	0.0000
3	70,664.300	70,872.609	70,780.451	0.668	0.0000
4	69,863.116	70,143.256	70,019.320	0.733	0.0000
5	69,479.188	69,831.159	69,675.444	0.666	0.0000
6	69,384.764	69,808.565	69,621.072	0.693	0.0001
7	69,368.135	69,863.767	69,644.496	0.728	0.0002

**Table 6:** Latent class coefficients for six-class model of vulnerable older adults.

Variable		Class 1	Class 2	Class 3	Class 4	Class 5	Class 6
X8	Someone to meeting/contacting	0.002	0.198	0.009	0.444	0.001	0.009
X9	Someone to discuss personal affairs	0.154	0.965	0.107	0.892	0.053	0.093
X10	Someone for help	0.009	0.695	0.037	0.826	0.014	0.020
X4	Health self-assessment	0.723	0.136	0.653	0.798	0.000	0.069
X5	Barthel index	0.505	0.000	0.307	0.485	0.030	0.098
X6	Chronic	0.965	0.575	0.944	0.934	0.626	0.627
X1	Income	0.998	0.575	0.516	0.894	0.305	0.994
X2	Economic independence	0.639	0.077	0.028	0.413	0.019	0.602
X3	Housing property	0.353	0.189	0.114	0.214	0.068	0.250
Proportion (%)		12.76	1.34	19.17	1.46	51.06	14.21

**Table 7:** Types of vulnerable older adults.

Vulnerability Degree	Type			
	Name	Classification	Characteristics	Proportion
High vulnerability	Multi-vulnerable	Class 4	Lack of social support, and poor health and economic status.	1.46%
Moderate vulnerability	Dual-vulnerable	Class 1	Common level of social support, while poor health and economic status.	12.76%
Slight vulnerability	Support-vulnerable	Class 2	Common level of health and economic status, while lack of social support.	1.34%
	Economy-vulnerable	Class 6	Common level of social support and health status, while poor economic status.	14.21%
	Health-vulnerable	Class 3	Common level of social support and economic status, while poor health status.	19.17%
Potential vulnerability	Potential-vulnerable	Class 5	Better level of social support, economic status, and health status.	51.06%

**Table 8:** Characteristics of different types of vulnerable older adults.

Variables	High/Moderate				Slight											
	Multi/Dual				Support				Economy				Health			
	All	NA-	A-	LR	All	NA-	A-	LR	All	NA-	A-	LR	All	NA-	A-	LR
Gender																
Male	33.7	24.1	35.7	***	64.6	75.9	55.6	*	40.2	22.8	44.0	***	48.7	49.0	49.5	ns
Female	66.3	75.9	64.3		35.4	24.1	44.4		59.8	77.2	56.0		51.3	51.1	50.5	
Age (years)																
60–69	35.2	38.5	35.1	ns	59.2	53.7	61.9	ns	44.1	44.4	43.6	ns	46.6	35.1	58.5	***
70–79	36.6	35.0	36.5		31.5	31.5	33.3		35.9	35.7	36.5		34.4	39.3	29.4	
80+	28.3	26.5	28.4		9.2	14.8	4.8		20.0	19.9	19.9		19.0	25.6	12.1	
Marital status																
No spouse	57.8	59.5	57.1	ns	35.4	33.3	34.9	ns	50.1	59.7	47.8	**	32.6	35.8	29.1	**
With spouse	42.2	40.5	42.9		64.6	66.7	65.1		49.9	40.3	52.2		67.4	64.2	70.9	
Living																
Alone	18.3	15.5	18.6	ns	20.0	11.1	25.4	ns	16.3	19.1	15.3	ns	14.0	14.3	13.9	ns
With non-descendants	21.1	20.5	21.3		23.1	27.8	22.2		20.9	18.6	21.6		38.1	36.9	39.0	
With descendants	60.6	64.0	60.1		56.9	61.1	52.4		62.8	62.3	63.2		47.9	48.8	47.2	
Social insurance																
Not participate	91.9	87.9	92.6	*	93.9	100.0	87.3	**	89.3	87.0	90.1	ns	94.3	97.3	90.5	***
Participate	8.1	12.1	7.4		6.2	0.0	12.7		10.7	13.0	9.9		5.7	2.7	9.6	
N	1384	200	1147		130	54	63		1383	216	1114		1865	812	880	

Note: (1) NA- stands for older adults with non-agricultural household registration, which means urban adults. A- stands for older adults with agricultural household registration, which means rural adults. (2) + *p* < 0.1, * *p* < 0.05, ** *p* < 0.01, *** *p* < 0.001.

## Data Availability

Publicly available datasets were analyzed in this study. This data can be found here: http://class.ruc.edu.cn (accessed on 2 October 2021).
